# The Effect of a Community-Based, Primary Health Care Exercise Program on Inflammatory Biomarkers and Hormone Levels

**DOI:** 10.1155/2014/185707

**Published:** 2014-07-17

**Authors:** Camila Bosquiero Papini, Priscila M. Nakamura, Lucas P. Zorzetto, Janice L. Thompson, Anna C. Phillips, Eduardo Kokubun

**Affiliations:** ^1^Department of Physical Education, São Paulo State University, Avenida 24-A, 1515 Bela Vista, 13506-900 Rio Claro, SP, Brazil; ^2^School of Sport, Exercise and Rehabilitation Sciences, University of Birmingham, Edgbaston, Birmingham, UK

## Abstract

The aim of this study was to analyze the impact of a community-based exercise program in primary care on inflammatory biomarkers and hormone levels. The 1-year quasiexperimental study involved 13 women (mean age = 56.8 ± 11.4 years) and it was developed in two basic health care units in Rio Claro City, Brazil. The physical exercise intervention was comprised of two, 60-minute sessions/week. The inflammatory biomarkers were measured at baseline, 6 months, and 1 year. Repeated measures ANOVA analyses indicated that the intervention was effective in reducing CRP and TNF*α* after 1 year compared to baseline and 6 months (*P* < 0.05). There were no changes in IL10, IL6, and insulin after 1 year. However, leptin significantly increased at 1 year (*P* = 0.016). The major finding of this study is that a community-based exercise program can result in a decrease or maintenance of inflammatory biomarkers after 1 year, and thus has the potential to be a viable public health approach for chronic disease prevention.

## 1. Introduction

It is well established that chronic diseases are the leading cause of mortality in the world. According to the World Health Organization [1] 60% of all death is attributed to cardiovascular diseases, diabetes, cancers, and chronic respiratory diseases. The inflammatory process related to chronic diseases, characterized by dysregulation in the balance between pro- and anti-inflammatory processes, is linked with several complications such as insulin resistance, endothelial dysfunction, atherosclerosis, and vascular and metabolic disorders [2–5].

Regular physical exercise has been increasingly viewed as an effective therapeutic strategy for the management of chronic diseases [6]. It has long been known that regular physical activity induces multiple adaptations within skeletal muscles and the cardiorespiratory system, providing positive outcomes for the prevention and treatment of chronic diseases [7, 8]. Some studies have indicated that regular physical activity has anti-inflammatory effects and is associated with improvement in inflammatory biomarkers such as a reduction in levels of the proinflammatory cytokines [9–14]. According to Pedersen [8], the anti-inflammatory processes provided by physical exercise play important roles in the protection against diseases associated with low-grade inflammation such as cardiovascular diseases and type 2 diabetes.

Considering that physical inactivity is the fourth leading cause of death worldwide [15] and causes 6–10% of the major noncommunicable diseases [6], it is necessary to induce social, economic, and environmental changes and multiple strategies that promote public policies related to physical active life style. “Saúde Ativa Rio Claro” (SARC) is a community-based exercise intervention in primary care designed to promote and maintain physical activity levels of residents in Rio Claro City, Brazil. Since 2001, SARC operates in basic health care units and reaches approximately 400 low-income adults aged 35 years or older [16]. Evidence suggests that this program improves blood cholesterol, LDL, HDL, and glucose profiles [17, 18]. However, it is unknown whether the SARC intervention can improve inflammatory biomarkers and thus potentially aid in the prevention of chronic disease and associated complications. Therefore, the aim of this study was to assess the impact of SARC on a range of common inflammatory biomarkers and hormone levels in adult women, including leptin, insulin, C-reactive protein (CRP), interleukin 6 (IL6), tumor necrosis factor alpha (TNF*α*), and interleukin 10 (IL10). It was hypothesized that there would be an increase in IL10 and a decrease in inflammatory markers (CRP, IL6, and TNF*α*) and hormone (leptin and insulin) levels after 1 year of SARC intervention.

## 2. Methods

### 2.1. Participants

**Figure 1:**
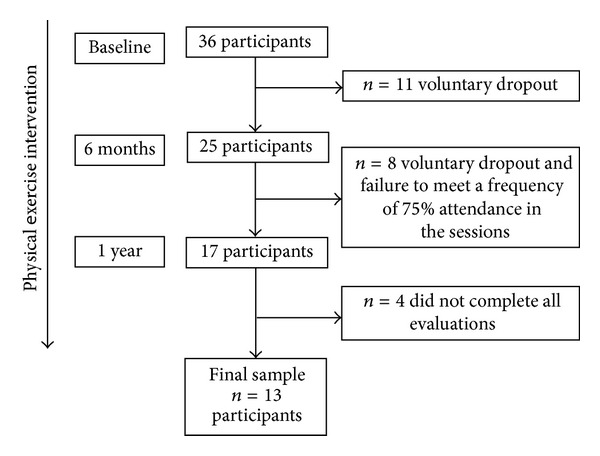
Recruitment of participants for the study. Evaluations were done at baseline, 6 months, and 1 year of SARC intervention.

This 1-year quasiexperimental study was developed in two basic health care units in Rio Claro City, Brazil. Adult females were recruited via flyers and newspaper advertisements. Participants were assigned to the intervention group based upon proximity from their residence. Thirty-six participants were recruited at the beginning of intervention. As a result of either voluntary dropout or failure to meet the inclusion criterion for the study (frequency of 75% attendance in the sessions), 25 participants remained in the intervention after 6 months. Although 17 participants completed the 1 year intervention, four participants did not complete all evaluations; thus the final sample size was 13 women (mean age = 56.8 ± 11.4 years, Figure 1). The study was approved by the Human Research Ethics Committee of Biosciences Institute, UNESP, protocol number: 2308.

### 2.2. Physical Exercise Intervention

SARC is a community-based exercise intervention comprised of two 60-minute sessions/week of physical exercises. The sessions were divided in warm-up activities (5 minutes), moderate intensity aerobic exercise (30 minutes), strength-training exercises (20 minutes), and cool-down activities (5 minutes). Furthermore, during each session, the participants received counseling designed to increase daily physical activity levels and encourage participation in physical exercise outside of the intervention.

The warm-up and cool-down activities included static stretching exercises and articular movements. Static stretching was maintained for a minimum period of 15 to 30 seconds, twice for each muscle group. The participants were advised to sustain a muscle stretch that did not cause pain [19, 20]. The aerobic exercises consisted of walking at moderate intensity (60–70% of peak heart rate). The target zone for exercise was calculated using the equation HRpeak = 206 − (0.88 × age), as suggested by Gulati et al. [21]. All participants were instructed to maintain a subjective effort between 13 and 15 [22] on the Borg scale [23] during walking. Four participants were randomly selected to measure the intensity of their activity twice a month using a cardiac rate monitor (Polar, FS1) and the subjective effort scale. The strength-training exercises were performed using free weights, exercise mats, and latex exercise bands. Exercises included all major muscle groups and were performed in 3 sets of 30 seconds, followed by one minute of recovery.

### 2.3. Inflammatory Biomarkers Measures

A 10 mL venous blood sample was collected at baseline, after 6 months, and after 1 year of intervention, in the morning after 12 hours of fasting. The blood sample was transported under refrigeration to the laboratory within 30 minutes, centrifuged for 10 minutes with the serum immediately separated following centrifugation. The inflammatory biomarkers were analyzed in duplicate using commercial kits. C-reactive protein (CRP) was analyzed using an enzyme-linked immunosorbent assay (ELISA). Interleukin 10 (IL10), interleukin 6 (IL6), tumor necrosis factor alpha (TNF*α*), leptin, and insulin were analyzed using Luminex technology assay (Luminex). Intra-assay coefficients were all <10%. To minimize analytical variations, the same technician tested all samples without changing reagent lots, standards, or control materials.

### 2.4. Statistical Analyses

Descriptive data are reported as means and standard deviations. The ratio of IL-10 to TNF-*α* (IL10/TNF-*α*) was calculated and compared in 1 year.

A repeated measures ANOVA was used to analyze the changes in anthropometric variables, inflammatory biomarkers, and hormones levels over time (baseline, 6 months, and 1 year). Significant differences were determined by Bonferroni post hoc tests. Statistical analyses were conducted using SPSS 20.0, with the alpha level set at *P* < 0.05.

## 3. Results

Table 1 shows the anthropometric characteristics of the participants (*n* = 13, mean age of 56.8 ± 11.4) at baseline, 6 months, and 1 year. No changes in weight, body mass index (BMI), or waist to hip ratio (WHR) were seen over time (*P* > 0.05). The prevalence of diseases was 7.7% (*n* = 1) for diabetes, 30.7% (*n* = 4) for obesity, and 38.5% for hypertension (*n* = 5). The prevalence of participants having at least 1 disease was 46.1% (*n* = 6).

**Figure 2:**
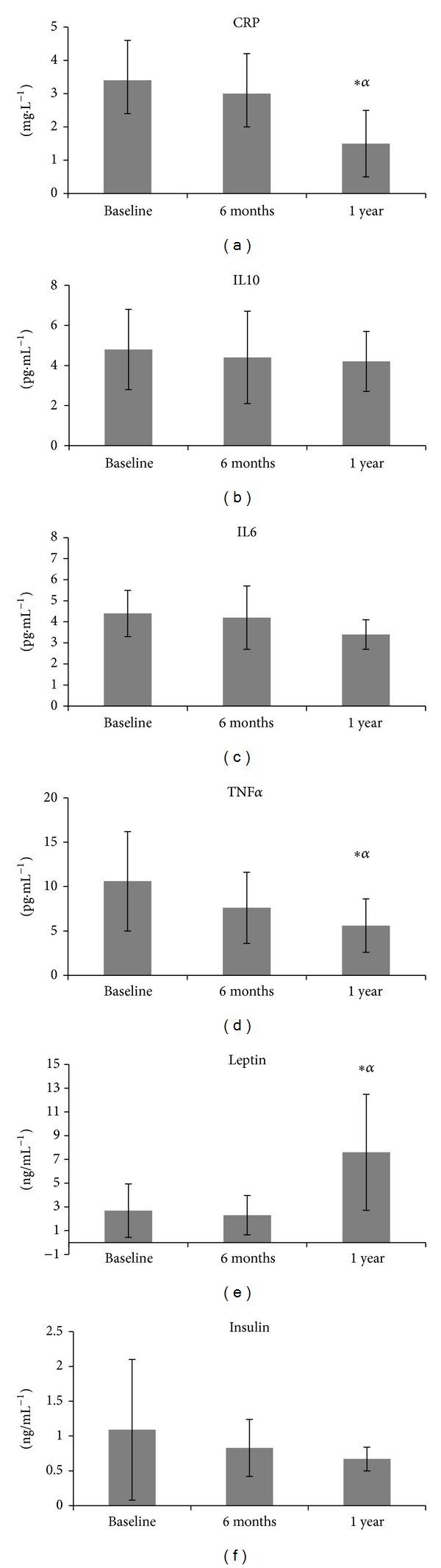
Levels of C-reactive protein (CRP), interleukin 10 (IL10), interleukin 6 (IL6), tumor necrosis factor alpha (TNF*α*), leptin, and insulin at baseline, after 6 months, and after 1 year of exercise intervention.  *Statistically significant difference from baseline.  ^*α*^Statistically significant difference from 6 months.

Table 2 and Figure 2 illustrate the inflammatory biomarkers and hormone concentration levels and indicate the outcomes of statistical analyses between time at baseline, 6 months, and 1 year. CRP levels significantly decreased after 1 year of intervention (1.5 ± 1.0 mg·L^−1^) compared to baseline (3.4 ± 1.2 mg·L^−1^, *P* = 0.001) and 6 months (3.0 ± 1.2 mg·L^−1^, *P* = 0.003). A significant decrease in TNF*α* levels was shown after 1 year of intervention (56.6 ± 3.0 pg·mL^−1^) compared to baseline (10.6 ± 5.6 pg·mL^−1^, *P* = 0.001) and 6 months (7.6 ± 4.0 pg·mL^−1^, *P* = 0.004). IL10, IL6, and insulin did not change over 1 year (*P* > 0.05). Leptin levels were significantly increased after 1 year (7.6 ± 4.89 pg·mL^−1^) of intervention compared to baseline (2.69 ± 2.25 pg·mL^−1^, *P* = 0.016) and 6 months (2.3 ± 1.66 pg·mL^−1^, *P* = 0.003). The IL10/TNF*α* ratio increased after 1 year of intervention (BL = 0.59 ± 0.4; 6 M = 0.64 ± 0.2; 1 Y = 0.85 ± 0.3).

## 4. Discussion

Chronic inflammation is an important pathophysiological factor in the development of several diseases and complications, through the effects of proinflammatory cytokines such as TNF*α* and IL6, among others [2–5]. On the contrary, anti-inflammatory cytokines, such as adiponectin and IL-10, seem to be protective against pathological conditions [24, 25].

Analyses indicate that the SACR intervention was effective in decreasing CRP and TNF*α* levels and maintaining IL10, IL6, and insulin levels over 1 year. However, leptin levels increased over 1 year. Several studies show that inflammatory biomarkers are reduced following longer term lifestyle modification involving reduced food intake and increased physical activity [9]. Thus, the effects of regular physical activity on basal levels of inflammatory markers have been used to recommend exercise as an anti-inflammatory therapy. According to Soares and de Souza [14] integrative interventions, including diet, moderate aerobic exercise (60% to 80% of HRmax or 50% to 60% of VO_2max⁡_) and circuit resistance training (8 to 10 exercises, 8 to 12 repetitions), health education, and counseling, used together, appeared to be effective strategies to improve inflammatory biomarkers in women.

Our results (Table 2 and Figure 2) indicated that SARC was effective in decreasing CRP levels after 1 year compared to baseline and 6 months. These findings are in agreement with other studies in the literature indicating that a physical lifestyle can reduce CRP levels [13, 26–29]. CRP has a long plasma half-life (>96 h), no variation of diurnal or seasonal, and no age or gender dependence [30, 31]. It plays a pivotal role in the innate immune response, is released in response to a variety of proinflammatory cytokines, and is triggered by many factors such as cardiovascular diseases, trauma, malignancy, and chronic arthritis [32]. In our study, the 56% decrease in CRP is clinically relevant because the value changed from a level considered “high risk” for cardiovascular disease at baseline (above 3.0 mg/L) to an “average risk” (1.0 to 3.0 mgL) after 1 year of the SARC intervention.

According to You et al. [33], findings about the relationship between physical exercise and inflammatory biomarkers are more consistent for CRP than for other biomarkers. However, the SARC intervention was effective in reducing TNF*α* (Table 2 and Figure 2) after 1 year compared to baseline and after 6 months. Studies have indicated that regular physical activity is associated with a reduction or no change in TNF*α* [27, 28, 34, 35]. TNF-*α* is a cytokine with a varied range of proinflammatory activities, such as influencing the atherosclerotic process both by causing metabolic perturbations and by increasing the expression of cellular adhesion molecules [36].

No changes were detected in IL10 following 1 year of intervention (Table 2). IL10 has multifaceted anti-inflammatory properties. It is able to reduce serum levels of TNF*α* and IL6 and plays a protective role against atherosclerosis [24, 25]. There is lack of consensus in the literature as to whether physical activity can improve IL10 levels. Kadoglou et al. [28] demonstrated in their study that a higher volume of aerobic exercise (four times/week, 45–60 min/session) was effective in increasing IL10 levels after 6 months. Similarly, Jankord and Jemiolo [37] compared groups performing different amounts of physical activity volume and concluded that the higher volume was associated with an increase of IL-10 [37]. Thus, it appears that 2 sessions per week of physical exercise delivered by SARC may be insufficient to improve IL10 levels. However, the IL10/TNF*α* ratio increased after 1 year of intervention. This result indicates that physical exercise was able to improve the proportion of anti- to proinflammatory cytokines after 1 year.

The SARC intervention did not change IL6 levels following 1 year of intervention (Table 2). Some studies have reported that physical exercise is correlated with lower IL6 levels [13, 29, 34, 37–40]. However, our results are in agreement with other studies. Olson et al. [41] found that an intervention consisting of at least two training sessions per week was not effective in reducing IL6 levels after 1 year. Campbell et al. [42] and Donges et al. did not also find lower levels of IL6 following physical exercise interventions [43]. Different cells produce IL6 and this cytokine plays both “good” and “bad” roles depending on the circumstances. It has been suggested that an elevation in IL6 in response to physical exercise can exert an anti-inflammatory role. Myokine, the IL6 from muscle, can increase during physical exercise. It wields metabolic effects on liver and adipose tissues (activating glycogenolysis and lipolysis) and inhibits the production of TNF*α* [44, 45]. On the other hand, IL6 is also secreted by macrophages and lymphocytes in response to injury or infection [46] and has been associated with several pathological conditions as a marker of low-grade inflammation [47, 48]. Thus, the maintenance of IL6 levels during a 1-year intervention could be considered a positive outcome.

It is currently well accepted that regular physical exercise is an effective therapeutic intervention to reduce the risk of developing insulin resistance by improving glucose tolerance and insulin action in individuals predisposed to developing type 2 diabetes [7]. It has been hypothesized that insulin resistance increases with age due to increased adiposity, decreased lean muscle mass, changes in dietary habits, and reduced physical activity [49]. Although there was not a statistically significant change in insulin in the present study (Table 2), insulin levels decreased by 38.4% after 1 year of the intervention, suggesting that insulin sensitivity may have improved, although an insulin sensitivity test in participants would be needed to confirm this.

In the present study, leptin levels were maintained until 6 months and then increased significantly after 1 year of the intervention (Table 2 and Figure 2). Despite these changes, leptin levels remained in the normal range (2.5–21.8 ng/mL). According to Mota and Zanesco [50], the relationship between physical activity and plasma leptin is unclear, with some studies showing a reduction in their levels while others fail to find any change. Recently, Akbarpour [13] demonstrated that 12 weeks of physical exercise was able to reduce leptin levels, BMI, and IL6, and in contrast to our findings they did not find any changes in TNF*α*.

Plasma levels of leptin can increase as the result of obesity [51]; in the present study we saw no changes in body weight, BMI, or WHR after 1 year. In addition, TNF-*α* and CRP have been shown to be related to high levels in adipose tissue, and its level in the circulation indicates the production of these biomarkers in adipose tissue [51]. In the present study, although no decrease in BMI and weight was observed, the levels of TNF*α* and CRP were decreased, supporting the effect of exercise on these biomarkers independent of weight loss. Current evidence supports that exercise training reduces chronic inflammation and this effect is independent of the exercise induced weight loss [33].

The mechanisms related to physical exercise as a therapy in changing inflammatory biomarkers are not clear, despite studies showing positive outcomes. The discrepancy between the results from various studies in the literature can be attributed to the differences among the groups studied, training period, volume, intensity, duration, and type of training.

This study has a number of limitations that should be considered. The small sample size that resulted in the study has low statistical power and was a result of the difficulty in maintaining a 75% participation rate in the intervention sessions over the 1-year intervention period. We attempted to reduce dropout by assigning participants to an intervention groups geographically near their home. In addition, this study employed a quasiexperimental design, and thus we are not able to state with confidence that the changes in inflammatory markers are due to participation in the SARC intervention. We attempted to include a control group (doing no physical exercise over 1 year) to allow us to conduct a controlled trial, but the university ethics committee would not approve this study design.

Considering the fact that 46.1% of participants already had at least 1 disease related with the inflammation process, this study illustrates that a public health exercise intervention delivered in low-income communities has the potential to exert a beneficial effect and improve or maintain inflammatory biomarkers profiles, assisting in the prevention of chronic diseases. However, a larger randomized controlled trial needs to be conducted to confirm or refute these suggestive findings.

## 5. Conclusion

The major finding of the present study was that a public health exercise intervention was effective in decreasing CRP and TNF*α* levels and maintaining IL10, IL6, and insulin levels over 1 year. Developing and delivering a community-based, public health exercise intervention like SARC could be a viable initiative to promote health at the public health level.

## Figures and Tables

**Table 1:** Anthropometric characteristics (mean, standard deviation) of participants at baseline, after 6 months, and after 1 year of exercise intervention.

	Baseline	6 months	1 year	*P* value BL versus 6 M	*P* value BL versus 1 Y	*P* value 6 M versus 1 Y
Weight (kg)	67.3 ± 11.5	66.8 ± 11.4	67.2 ± 10.9	0.541	0.631	1.000
BMI (kg/m^2^)	27.5 ± 5.6	26.8 ± 6.0	27.9 ± 5.6	0.500	0.316	1.000
WHR	0.88 ± 0.8	0.86 ± 0.8	0.89 ± 0.7	0.863	0.326	1.000

BL: baseline; 6 M: 6 months; 1 Y: 1 year; BMI: body mass index; WHR: waist and hip ratio.

**Table 2:** Inflammatory biomarkers and hormone concentration levels (mean, standard deviation) at baseline, after 6 months, and after 1 year of exercise intervention.

Biomarker	BL	6 M	1 Y	*P* value BL versus 6 M	*P* value BL versus 1 Y	*P* value 6 M versus 1 Y
CRP (mg*·*L^−1^)	3.4 ± 1.2	3.0 ± 1.2	1.5 ± 1.0^∗*α*^	0.999	0.001	0.003
IL10 (pg*·*mL^−1^)	4.8 ± 2.0	4.4 ± 2.3	4.2 ± 1.5	0.988	0.681	0.602
IL6 (pg*·*mL^−1^)	4.4 ± 1.1	4.2 ± 1.5	3.4 ± 0.7	0.999	0.236	0.163
TNF*α* (pg*·*mL^−1^)	10.6 ± 5.6	7.6 ± 4.0	5.6 ± 3.0^∗*α*^	0.082	0.001	0.004
Leptin (ng/mL^−1^)	2.69 ± 2.25	2.30 ± 1.66	7.60 ± 4.89^∗*α*^	0.999	0.016	0.003
Insulin (ng/mL^−1^)	1.09 ± 1.01	0.83 ± 0.41	0.67 ± 0.17	0.898	0.405	0.642
IL10/TNF*α*	0.59 ± 0.4	0.64 ± 0.2	0.85 ± 0.3	—	—	—

BL: baseline; 6 M: 6 months; 1 Y: 1 year; CRP: C-reactive protein; IL10: interleukin 10; IL6: interleukin 6; TNF*α*: tumor necrosis factor alpha.

∗Statistically significant difference from baseline.

^*α*^Statistically significant difference after 6 months.
